# Creating Walkable Communities: Understanding Trade-Offs

**DOI:** 10.5888/pcd15.180123

**Published:** 2018-08-30

**Authors:** Susan A. Carlson, John D. Omura, Kathleen B. Watson, Janet E. Fulton

**Affiliations:** 1 Division of Nutrition, Physical Activity, and Obesity, Centers for Disease Control and Prevention, Atlanta, Georgia

## Abstract

Implementing community design strategies can offer benefits related to walkability; however, they may also come with trade-offs to other community needs and desires. We examined public sentiment for 2 trade-offs among 2014 SummerStyles survey respondents (n = 3,995). About 33% of adults reported strongly favoring safer street design even if driving is slower; only 19% reported strongly favoring community design with walkable destinations even if homes are closer together. Walking frequency was positively associated with strongly favoring trade-offs, while differences by other demographic characteristics depended on the trade-off. Addressing public sentiment for potential trade-offs may be important when promoting walkable design strategies.

## Objective

Walking is an easy way for Americans to start and maintain a physically active lifestyle to achieve health benefits (1,2). Walking, along with other physical activities, can be promoted through community design strategies (2–4). For example, policies and practices can encourage mixed land use to improve the diversity and proximity of community destinations, and street design can be enhanced to include features, such as sidewalks and crosswalks, to improve safety (3,4).

Implementing supportive community design strategies can result in benefits related to walkability (eg, improved safety, easier access to destinations). However, they may come with consequences (eg, decreased vehicle speed, increased residential density). It is important to understand public sentiment related to trade-offs between benefits and consequences because this can influence public support for these strategies. Strong public support can be an important factor in successfully implementing strategies to create walkable communities (5). First, we examine public sentiment among US adults for 2 potential trade-offs: 1) designing streets with features to make walking safer even if driving is slower and 2) designing communities with stores and other places within walking distance even if homes are built closer together. Second, we examine whether strong positive sentiment (ie, strongly favor) for these trade-offs differs by demographic characteristics and walking frequency.

## Methods

### Survey

The 2014 Porter Novelli ConsumerStyles database is built from a series of web-based surveys via the GfK KnowledgePanel that gather insights about US consumers, including information about health attitudes and behaviors. The panel has about 55,000 panelists randomly recruited through probability-based sampling. The initial SpringStyles survey was sent to a random sample of adult panelists (aged ≥18 years) and a supplemental sample of panelists with children.

We used data from the 2014 SummerStyles survey that was sent during June and July to 6,159 adults who had previously completed the SpringStyles survey. The survey took approximately 36 minutes to complete and 4,269 surveys were returned (response rate, 69%). Respondents were not required to answer any of the questions and could exit the survey at any time. Those who completed the survey received reward points worth approximately $10 and were eligible to win in-kind prizes through monthly sweepstakes.

### Measures

To assess sentiment related to trade-offs, respondents were asked if they favor or oppose (strongly oppose, oppose, favor, strongly favor, and don’t know)

1. Designing streets with sidewalks, crosswalks, stop signs, and other features to make it safer to walk, even if it means driving slower.
2. Designing communities so that more stores and other places are within walking distance of homes, even if this means building homes closer together.

Respondents were asked how often they usually walk for at least 10 minutes at a time. Adults who indicated that they were physically able to walk were categorized into 1 of 3 walking frequency categories: frequently (every day or most days), sometimes (some days), and rarely (hardly ever or never). Demographic characteristics were sex, age group, education, race/ethnicity, census region (6), metropolitan statistical area (MSA) status (7), and home ownership.

### Statistical analysis

We examined public sentiment for each trade-off overall. Adjusted logistic regression analyses were used to examine the association between walking frequency and demographic characteristics with reporting strongly favoring each trade-off. Analyses were conducted by using SUDAAN, Release 11 (Research Triangle Institute) to account for survey weights.

## Results

About 82.2% of adults reported favoring (48.9%) or strongly favoring (33.3%) safer street design even if driving is slower (Figure). About 55.7% of adults reported favoring (36.3%) or strongly favoring (19.4%) community design with stores and other destinations within walking distance even if homes are closer together.

**Figure:**
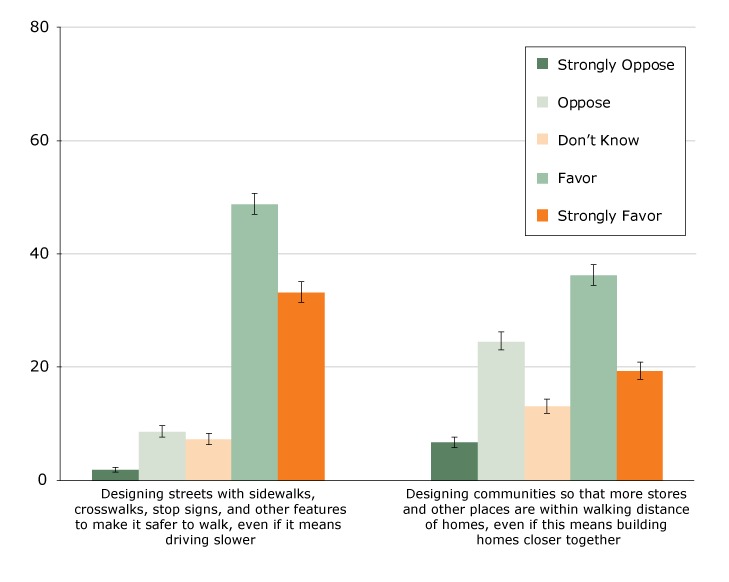
Percentage reporting level of support for trade-offs to create walkable communities among adults, SummerStyles 2014 (n = 3,995). Error bars represent the upper and lower bounds of the 95% confidence interval. Of the 4,269 respondents, 274 were excluded for missing data (n = 78) or because they indicated they were unable to walk when asked about how often they usually walk for at least 10 minutes at a time (n = 196). **Table:** 

Level of Support	Trade-off	
	Designing streets with sidewalks, crosswalks, stop signs, and other features to make it safer to walk, even if it means driving slower, %	Designing communities so that more stores and other places are within walking distance of homes, even if this means building homes closer together, %
Strongly oppose	1.9 (1.4–2.4)	6.7 (5.8–7.7)
Oppose	8.6 (7.6–9.7)	24.6 (23.1–26.2)
Don’t know	7.3 (6.4–8.3)	13.1 (11.8–14.4)
Favor	48.9 (47.1–50.8)	36.3 (34.5–38.1)
Strongly favor	33.3 (31.5–35.1)	19.4 (17.9–20.9)

Prevalence of strongly favoring each trade-off was significantly higher for adults who reported frequent walking compared with walking sometimes or rarely (Table). In addition, significant differences in prevalence of strongly favoring safer street design even if driving is slower were found by race/ethnicity and census region, while significant differences in the prevalence of strongly favoring community design with walkable destinations even if homes are built closer together were found by age, education, race/ethnicity, and MSA status. In adjusted models, findings were similar.

**Table:** Prevalence and Adjusted Odds Ratio of Strongly Favoring Trade-offs to Create Walkable Communities, by Select Characteristics Among Adults, SummerStyles 2014^a^

Characteristic	n	Strongly Favored			
		Designing Streets With Sidewalks, Crosswalks, Stop Signs, and Other Features to Make it Safer to Walk, Even if it Means Driving Slower		Designing Communities so That More Stores and Other Places Are Within Walking Distance of Homes, Even if This Means Building Homes Closer Together	
		%^b^ (95% CI)	AOR^c^ (95% CI)	%^b^ (95% CI)	AOR^c^ (95% CI)
**Total**	3,995	33.3 (31.5–35.1)	NA	19.4 (17.9–20.9)	NA
**Walking frequency**					
Frequently	1,383	40.5 (37.5–43.6)^x^	1.66 (1.34–2.05)	24.0 (21.3–26.8)^x^	1.68 (1.29–2.18)
Sometimes	1,629	29.9 (27.3–32.7)^y^	1.02 (0.82–1.26)	18.3 (16.1–20.7)^y^	1.18 (0.91–1.54)
Rarely	983	29.2 (25.9–32.8)^y^	1 [Reference]	15.1 (12.6–18.0)^y^	1 [Reference]
**Sex**					
Male	1,959	31.9 (29.5–34.5)	0.88 (0.75–1.04)	19.9 (17.9–22.2)	1.06 (0.87–1.29)
Female	2,036	34.6 (32.1–37.1)	1 [Reference]	18.9 (16.9–21.0)	1 [Reference]
**Age, y**					
18–34	689	32.3 (28.5–36.3)	0.80 (0.63–1.03)	22.2 (19.0–25.8)^x^	1.40 (1.03–1.89)
35–49	1,118	33.0 (29.7–36.4)	0.85 (0.67–1.06)	21.2 (18.4–24.3)	1.23 (0.93–1.63)
50–64	1,355	33.6 (30.8–36.6)	0.89 (0.72–1.10)	16.5 (14.3–18.8)^y^	0.96 (0.73–1.25)
≥65	833	35.0 (31.4–38.7)	1 [Reference]	16.5 (13.8–19.6)	1 [Reference]
**Education**					
High school graduate or less	1,400	30.6 (27.7–33.6)	0.82 (0.67–1.00)	15.0 (12.9–17.5)^x^	0.56 (0.44–0.71)
Some college	1,253	34.7 (31.6–38.0)	0.97 (0.80–1.18)	19.1 (16.5–21.9)^x^	0.69 (0.55–0.87)
College graduate	1,342	35.6 (32.6–38.7)	1 [Reference]	25.7 (23.0–28.6)^y^	1 [Reference]
**Race/ethnicity**					
White, non-Hispanic	3,008	30.9 (29.0–32.9)^x^	1 [Reference]	17.7 (16.2–19.4)^x^	1 [Reference]
Black, non-Hispanic	378	39.0 (33.5–44.7)^y^	1.35 (1.04–1.76)	19.6 (15.6–24.5)	1.16 (0.84–1.60)
Other	609	37.4 (32.9–42.1)^y^	1.37 (1.09–1.72)	24.2 (20.3–28.6)^y^	1.38 (1.06–1.80)
**MSA status^d^**					
Nonmetro	622	29.7 (25.5–34.2)	0.91 (0.72–1.14)	12.7 (9.8–16.3)^x^	0.66 (0.48–0.90)
Metro	3,373	33.9 (32.0–35.9)	1 [Reference]	20.6 (19.0–22.3)^y^	1 [Reference]
**Census region**					
Northeast	712	34.6 (30.5–38.9)^x^	1.38 (1.07–1.77)	18.8 (15.6–22.4)	1.06 (0.78–1.43)
Midwest	1,013	26.6 (23.5–30.0)^y^	1 [Reference]	16.3 (13.8–19.1)	1 [Reference]
South	1,410	37.3 (34.3–40.4)^x^	1.56 (1.26–1.94)	20.4 (18.0–23.1)	1.25 (0.97–1.62)
West	860	32.2 (28.4–36.1)	1.16 (0.90–1.49)	21.1 (17.9–24.7)	1.14 (0.84–1.53)
**Home ownership**					
Homeowner	3,049	32.6 (30.6–34.6)	0.90 (0.74–1.09)	19.6 (17.9–21.3)	1.13 (0.89–1.44)
Nonhomeowner	946	35.3 (31.7–39.0)	1 [Reference]	18.9 (16.1–22.0)	1 [Reference]

Abbreviations: AOR, adjusted odds ratio; CI, confidence interval; MSA, metropolitan statistical area status; NA, not applicable.

a Of 4,269 respondents, 274 were excluded for missing data (n = 78) or because they indicated they were unable to walk when asked about how often they usually walk for at least 10 min at a time (n = 196). Estimates were weighted using survey weights provided as part of the data set. Weights were created to match US Current Population Survey proportions for sex, age, household income, race/ethnicity, household size, education, region, MSA status, and internet access before joining the panel.

b Superscript x and y indicate significant differences: within subgroups, values that have a different letter are significantly different (Bonferroni corrected *P* < .05).

c Models adjusted for walking frequency, sex, age group, education, race/ethnicity, census region, MSA status, and home ownership.

d An MSA was categorized as metro if it was associated with at least 1 urbanized area that has a population of at least 50,000.

## Discussion

One in 3 adults strongly favored the trade-off of safer street design even if driving is slower, while only 1 in 5 adults strongly favored community design with walkable destinations even if homes are built closer together. Walking frequency was positively associated with strongly favoring both trade-offs, while differences by demographic characteristics generally depended on the trade-off. To garner public support for creating walkable communities, it may be important to address public sentiment for trade-offs and how this may vary in communities.

We found differences by demographic characteristics in public sentiment for trade-offs consistent with studies examining similar concepts. For example, we found that younger adults (aged 18–49) were more likely than older adults to strongly favor community design with walkable destinations even if homes are built closer together. Similarly, another study found that millennials (aged 18–34) were more interested in being within easy walking distance of destinations than other generations (8). We also found that nonwhites were generally more likely to support trade-offs than whites, although where differences were significant differed some based on the trade-off. Another study similarly found that nonwhites were more supportive of “activity-friendly communities” (9). Obtaining information about public sentiment related to a wider breadth of trade-offs may help identify which are most favored by different populations.

This study has strengths and limitations. One limitation was sample selection bias that may be associated with data from a panel survey of volunteers. However, previous research comparing random-digit–dialed and panel approaches has found a general equivalence between results, suggesting that findings from panel studies are as acceptable as those using respondents selected randomly for telephone surveys (10). A strength was that the sample was drawn from a large nationwide sample, allowing us to look at differences by many demographic characteristics.

Addressing public sentiment for trade-offs may be important when promoting walkable community design strategies, and this may vary by community. Findings from this study can help local practitioners better understand public sentiment for trade-offs when implementing strategies to create more walkable communities.

## References

[1] US Department of Health and Human Services. 2008 Physical activity guidelines for Americans. Washington (DC): US Department of Health and Human Services; 2008.

[2] US Department of Health and Human Services. Step It Up! The Surgeon General's Call to action to promote walking and walkable communities. Washington (DC): US Department of Health and Human Services, Office of the Surgeon General; 2015.

[3] Heath GW , Brownson RC , Kruger J , Miles R , Powell KE , Ramsey LT . The effectiveness of urban design and land use and transport policies and practices to increase physical activity: a systematic review. J Phys Act Health 2006;3(s1):S55–76. 10.1123/jpah.3.s1.s55 28834525

[4] Community Preventive Services Task Force. Physical activity: built environment approaches combining transportation system interventions with land use and environmental design. 2017. https://www.thecommunityguide.org/findings/physical-activity-built-environment-approaches. Accessed May 9, 2017.

[5] Sallis JF , Cervero RB , Ascher W , Henderson KA , Kraft MK , Kerr J . An ecological approach to creating active living communities. Annu Rev Public Health 2006;27(1):297–322. 10.1146/annurev.publhealth.27.021405.102100 16533119

[6] US Census Bureau. Geographic terms and concepts: census divisions and census regions. 2018. https://www.census.gov/geo/reference/gtc/gtc_census_divreg.html. Accessed January 4, 2018.

[7] US Census Bureau. Geographic terms and concepts: core based statistical areas and related statistical areas. 2018. https://www.census.gov/geo/reference/gtc/gtc_cbsa.html#mesa. Accessed January 4, 2018.

[8] National Association of Realtors. Community and Transportation Preferences Survey. U.S. metro areas, 2015. https://www.nar.realtor/sites/default/files/reports/2015/nar-psu-2015-poll-report.pdf. Accessed December 18, 2017.

[9] Librett JJ , Yore MM , Schmid TL , Kohl HW 3d . Are self-reported physical activity levels associated with perceived desirability of activity-friendly communities? Health Place 2007;13(3):767–73. 10.1016/j.healthplace.2006.07.003 16935021

[10] Fisher L , Kane N. Consumer panelist versus random digit dial respondent performance revisited: how similar and how different? Research on Research Report 64. Chicago (IL): Synovate Inc; 2004.

